# Enhancement in Corneal Permeability of Dissolved Carteolol by Its Combination with Magnesium Hydroxide Nanoparticles

**DOI:** 10.3390/ijms19010282

**Published:** 2018-01-17

**Authors:** Noriaki Nagai, Sakie Yamaoka, Yuya Fukuoka, Miyu Ishii, Hiroko Otake, Kazutaka Kanai, Norio Okamoto, Yoshikazu Shimomura

**Affiliations:** 1 Faculty of Pharmacy, Kindai University, 3-4-1 Kowakae, Higashi-Osaka, Osaka 577-8502, Japan; yamaoka0207@gmail.com (S.Y.); 1411710044j@kindai.ac.jp (Y.F.); 1411710022r@kindai.ac.jp (M.I.); hotake@phar.kindai.ac.jp (H.O.); 2 Department of Small Animal Internal Medicine, School of Veterinary Medicine, University of Kitasato, Towada, Aomori 034-8628, Japan; kanai@vmas.kitasato-u.ac.jp; 3 Department of Ophthalmology, Faculty of Medicine, Kindai University, 377-2 Ohno-Higashi, Osaka-Sayama, Osaka 589-8511, Japan; eyedoctor9@msn.com (N.O.); yoshis@med.kindai.ac.jp (Y.S.)

**Keywords:** carteolol, nanoparticle, magnesium hydroxide, glaucoma, corneal penetration

## Abstract

We prepared magnesium hydroxide (MH) nanoparticles, and investigated their effect when combined with dissolved carteolol on the bioavailability and intraocular pressure (IOP)-reducing effect of carteolol. The carteolol was solved in saline containing additives (0.5% methylcellulose, 0.001% benzalkonium chloride, 0.5% mannitol; CRT-solution). MH nanoparticles were prepared by a bead mill method with additives. Then carteolol/MH microparticle and carteolol/MH nanoparticle fixed combinations (mCMFC and nCMFC) were prepared by mixing the CRT-solution and MH particles. The transcorneal penetration and IOP-reducing effect of carteolol was evaluated in rabbits. The mean particle size of mCMFC was 7.2 μm, and the particle size was reduced to 73.5–113.5 nm by the bead mill treatment. The MH particles in nCMFC remained in the nano size range for 8 days after preparation, and the amounts of lacrimal fluid and corneal damage were unchanged by repetitive instillation of nCMFC (twice a day for 4 weeks). The transcorneal penetration of carteolol was enhanced by the combination with MH nanoparticles, and the IOP-reducing effect of nCMFC was significantly higher than that of CRT-solution or mCMFC. In conclusion, we designed nCMFC, and showed that the high levels of dissolved carteolol can be delivered into the aqueous humor by the instillation of nCMFC. Combination with MH nanoparticles may achieve an enhancement of corneal penetration for water-soluble drugs. These findings provide significant information that can be used to design further studies aimed at developing anti-glaucoma eye drugs.

## 1. Introduction

Glaucoma is an acquired disease that results in characteristic changes in the optic nerve head, and leads to vision impairment and blindness [1,2,3,4,5,6]. The main risk factor for glaucoma is considered to be long-term elevated intraocular pressure (IOP), and the regulation of the enhanced IOP is the most direct method of therapy for glaucoma. Treatment with topical eye drops is considered a first choice of therapy for glaucoma, and medications such as β-blockers, prostaglandin analogs, adrenergic agonists, sympathomimetics, carbonic anhydrase inhibitors, α_2_ adrenergic agonists, and α_1_-blockers are all used clinically [1,2,3,4,5,6]. In particular, β-blockers and prostaglandin analogs have a reputation for lowering IOP more than other drug classes.

Carteolol hydrochloride (carteolol) is a nonselective β-adrenoceptor antagonist, and also has endothelium-dependent vascular-relaxant effects [7] and intrinsic sympathomimetic activity [8,9]. It has been reported that carteolol can ameliorate retrobulbar hemodynamics in patients with primary open-angle glaucoma [10,11,12], and improves ocular perfusion of the optic nerve head in healthy humans [10,13,14]. Moreover, a combination of latanoprost and carteolol has been introduced, and has been widely used for many years as an ophthalmic solution for the treatment of glaucoma with simple ocular hypertension. On the other hand, carteolol when instilled has low bioavailability, since it is water-soluble, and thus has difficulty penetrating the hydrophobic cornea epithelium. Therefore, the remaining drug that enters the systemic circulation can cause bronchial asthma via antagonism of β-adrenoceptors. From these reasons, improvement of carteolol bioavailability is expected.

Recently, drug delivery systems (DDS) using nanoparticles have been investigated for their effectiveness in the management of glaucoma, and we have designed ophthalmic formulations containing solid nanoparticles using cilostazol [15,16,17], disulfiram [18,19], indomethacin [20], dexamethasone [21], and tranilast [22]. Moreover, we have shown that these ophthalmic formulations have high bioavailability [15,16,17,18,19,20,21,22]. We have also found that the co-instillation of magnesium hydroxide (MH, brucite) nanoparticles enhances the corneal penetration of dissolved timolol without observable corneal stimulation or obstruction of the nasolacrimal duct [23]. These studies suggest that combining MH nanoparticles with water-soluble drugs may be a useful way to improve drug bioavailability in the ophthalmic field. In this study, we prepared MH nanoparticles by the bead mill method, and investigated whether the combination of dissolved carteolol and MH nanoparticles enhances the bioavailability and IOP-reducing effect of carteolol.

## 2. Results

### 2.1. Changes in the Ocular Surface by the Instillation of Carteolol/MH Microparticle and Carteolol/MH Nanoparticle Fixed Combinations

**Figure 1:**
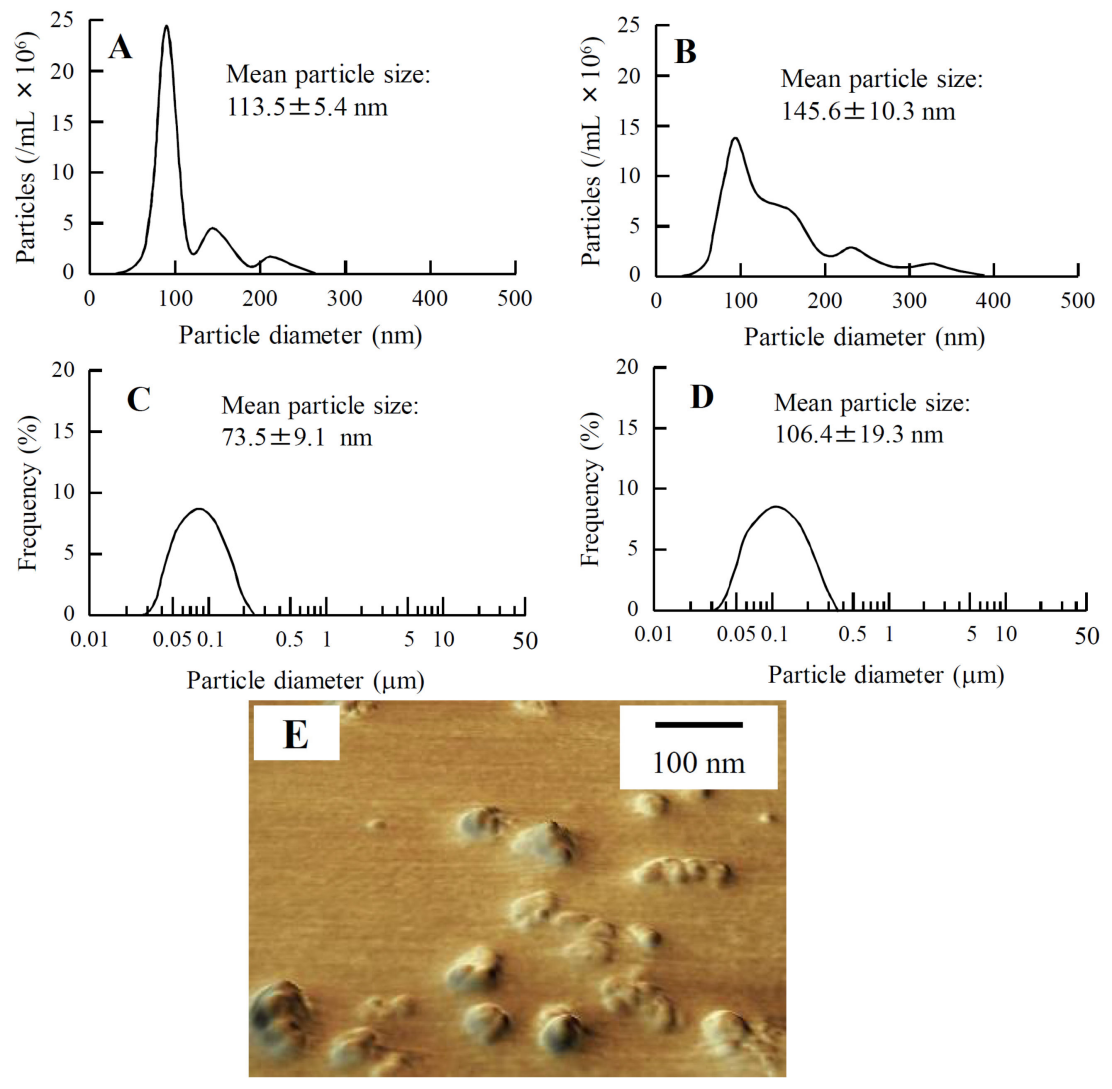
Size frequency distribution of the MH particles in nCMFC just after the bead mill treatment (**A**,**C**) or 8 days after bead mill treatment (**B**,**D**). The size frequency distributions were measured by using the NANOSIGHT LM10 (QuantumDesign Japan, Tokyo, Japan) (**A**,**B**) and SALD-7100 (**C**,**D**); (**E**) shows the scanning probe microscopy (SPM) image of nCMFC just after bead mill treatment. The MH particles in nCMFC remained in the nano size range for 8 days after bead mill treatment.

Figure 1 shows the changes in the size frequency distribution of carteolol/MH nanoparticle fixed combination (nCMFC) after treatment with the bead mill. The mean particle size of carteolol/MH microparticle fixed combination (mCMFC) was 7.2 μm, and the particle size was reduced to 73.5–113.5 nm by bead mill treatment (Figure 1A,C,E). nCMFC is stable for at least 8 days, since the particle size remained in the nano range for 8 days after preparation (Figure 1B,D).

**Figure 2:**
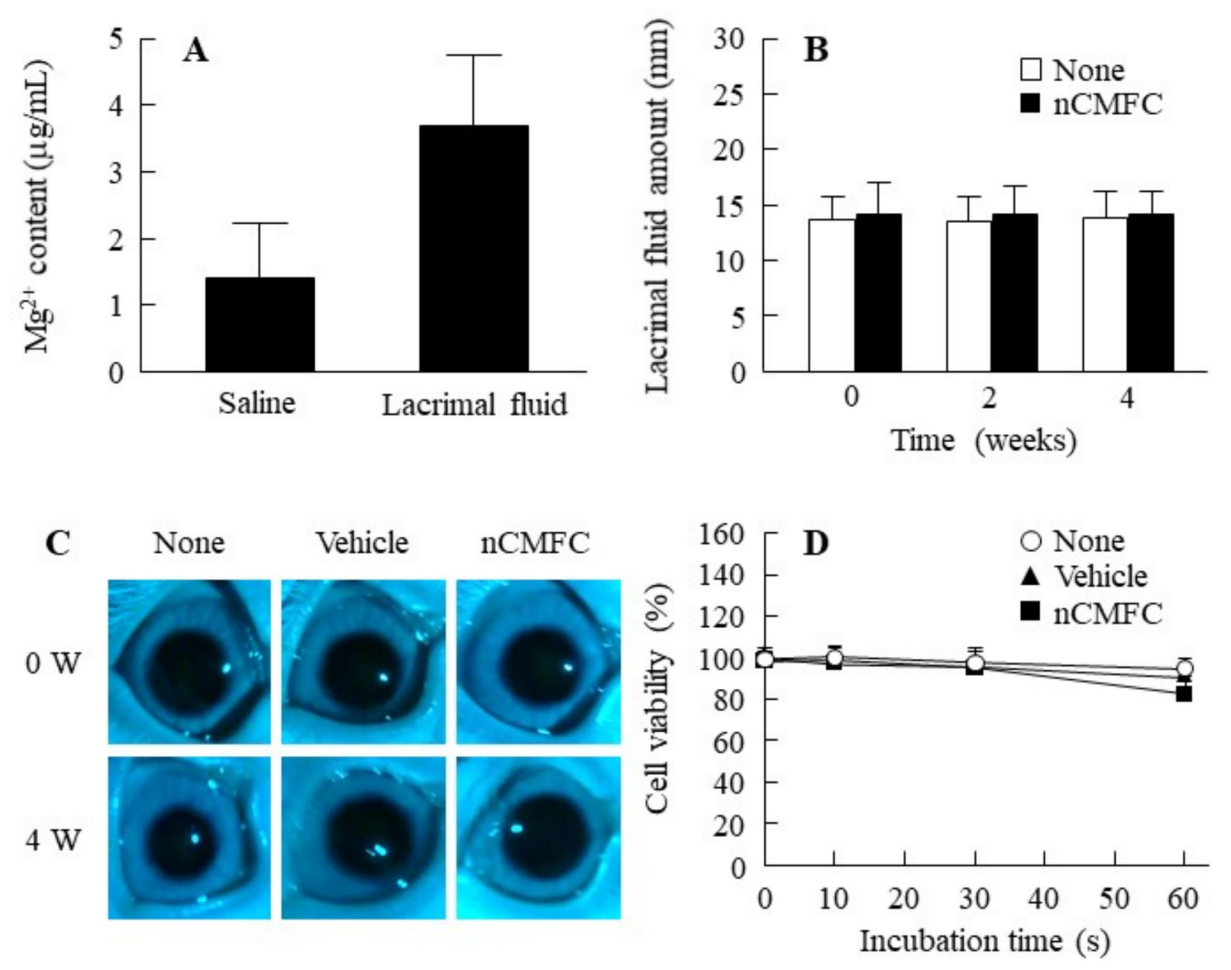
Effect of nCMFC on rabbit eyes. (**A**) Solubility of MH in nCMFC in saline and lacrimal fluid; (**B**) changes in amount of lacrimal fluid after repetitive instillation of nCMFC for 4 weeks (twice/day); (**C**) image of rabbit eye after the repetitive instillation of nCMFC for 4 weeks (twice/day); (**D**) viability of human corneal epithelial cell line cells (HCE-T) cells treated with nCMFC. None, non-treated groups. Vehicle, vehicle-instilled groups. nCMFC, nCMFC-instilled groups. *n* = 6–12. Solubility of MH in nCMFC in lacrimal fluid was low (0.00037%), and the behavior of lacrimal fluid (balance of secretion and excretion) was similar between rabbit eyes instilled with or without nCMFC. In addition, the corneal toxicity of nCMFC was not observed in the rabbit cornea and HCE-T cells.

Figure 2 shows the solubility of MH in lacrimal fluid (Figure 2A) and changes in lacrimal fluid after the repetitive instillation of nCMFC (Figure 2B). The solubility of MH in lacrimal fluid was somewhat greater than in saline; however, the MH was only marginally soluble in nCMFC (Mg^2+^; 3.7 μg/mL), and the ratio of Mg^2+^ in lacrimal fluid was 3.7 (ion, Mg^2+^):96.3 (solid, Mg) (Figure 2A), and the MH particle size in nCMFC was 121.5 ± 7.9 nm in the lacrimal fluid (nCMFC:lacrimal fluid = 1:1, the particle size was measured by NANOSIGHT LM10). In addition, the amount of lacrimal fluid was not changed by the repetitive instillation of nCMFC (Figure 2B). Figure 2C shows the effect of the repetitive instillation of nCMFC on rabbit cornea. No corneal damage was observed following the repetitive instillation of nCMFC for 4 weeks. The cell viability in human corneal epithelial cell line cells (HCE-T cells) treated with nCMFC was also similar to the non-treated HCE-T cells (Figure 2D).

### 2.2. Enhancement of Transcorneal Penetration and IOP-Reducing Effect of Carteolol in Combination with MH Nanoparticles

**Figure 3:**
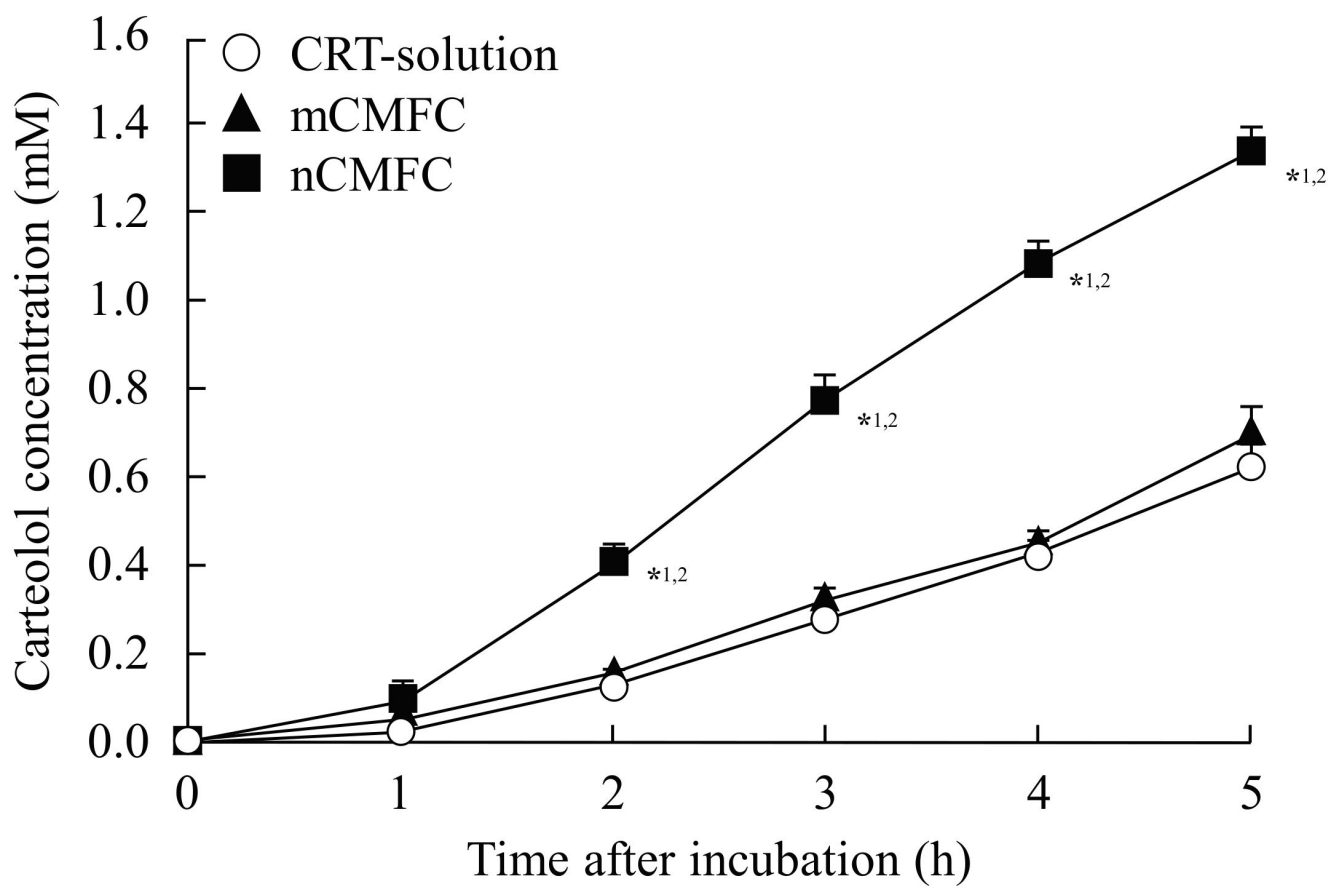
In vitro transcorneal penetration of carteolol from nCMFC. CRT-solution (○), carteolol solution-treated cornea. mCMFC (▲), mCMFC-treated cornea. nCMFC (■), nCMFC-treated cornea. *n* = 7. *^1^ *p* < 0.05, vs. CRT-solutions for each category. *^2^ *p* < 0.05, vs. mCMFC for each category. Although the combination with MH microparticles did not affect the transcorneal penetration of carteolol, the transcorneal penetration rate of carteolol was enhanced by its combination with MH nanoparticles.

**Figure 4:**
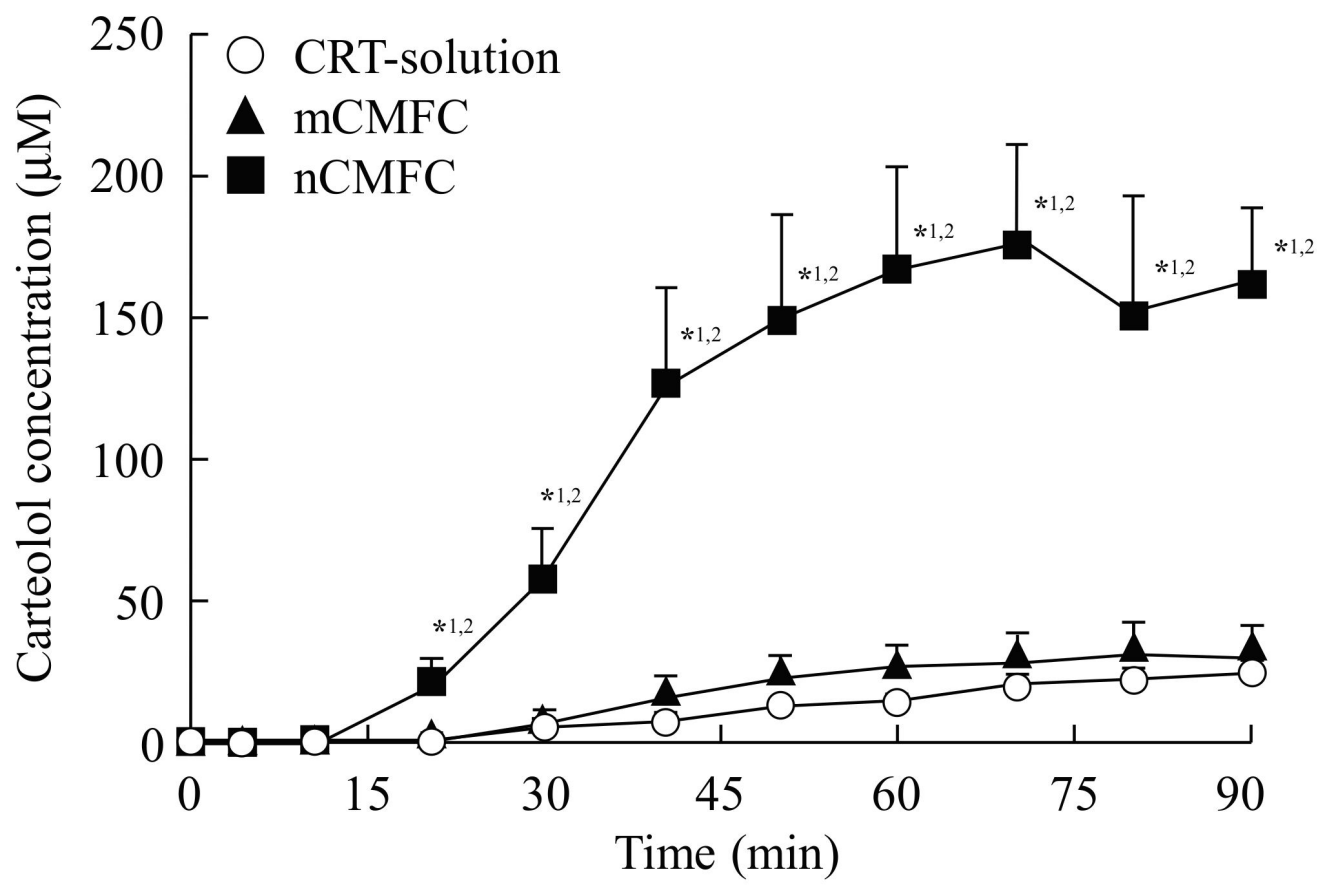
In vivo corneal permeability of carteolol from nCMFC. CRT-solution (○), CRT solution-instilled rabbit. mCMFC (▲), mCMFC-instilled rabbit. nCMFC (■), nCMFC-instilled rabbit. *n* = 7. *^1^ *p* < 0.05, vs. CRT-solution for each category. *^2^ *p* < 0.05, vs. mCMFC for each category. The carteolol concentration in the aqueous humor of rabbits instilled with nCMFC was significantly higher than in the aqueous humor of rabbits instilled with CRT-solution or mCMFC.

Figure 3 and Figure 4 show the in vitro (Figure 3) and in vivo (Figure 4) transcorneal penetration of carteolol after the instillation of CMFC, and Table 1 and Table 2 summarize the pharmacokinetic parameters calculated from the in vitro and in vivo transcorneal penetration data, respectively. Although there were no significant differences in the amount of penetration between the CRT-solution and mCMFC, the carteolol concentration in the aqueous humor was increased by combination with MH nanoparticles. Transcorneal penetration, *J*_c_, *K*_p_, and *D* values following treatment with nCMFC were all significantly higher than following the instillation of CRT-solution or mCMFC, and the area under the carteolol concentration-time curve (AUC) for nCMFC was 9.64-fold higher than that for the CRT-solution. On the other hand, the mean residence time (MRT) values showed no differences for the CRT-solution, mCMFC, or nCMFC.

**Figure 5:**
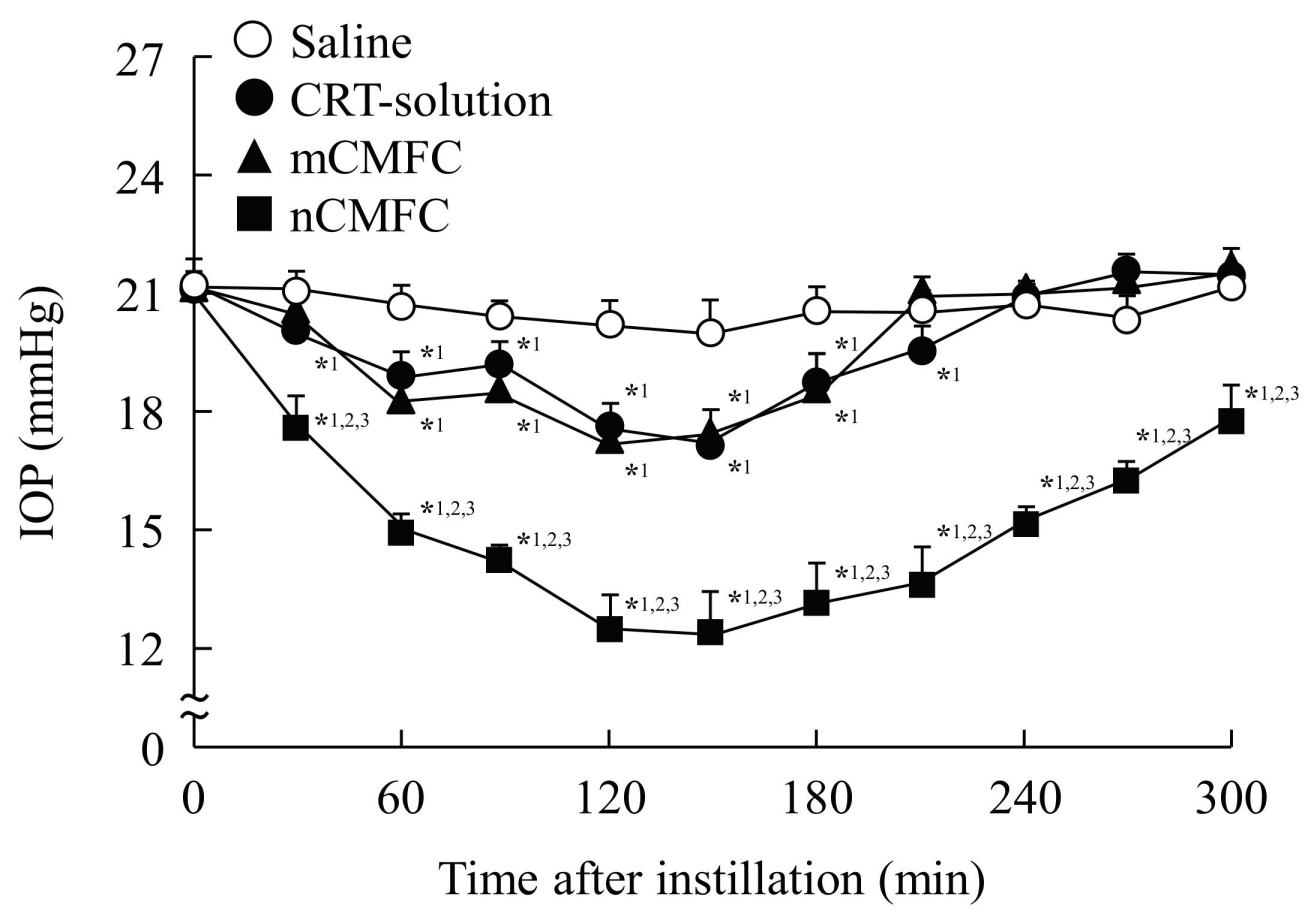
Preventive effect of nCMFC on the increased IOP in a rabbit model. Rabbits were kept in a dark room for 5 h before the experiment. Saline (○), saline-instilled rabbit. CRT-solution (●), CRT-solution-instilled rabbit. mCMFC (▲), mCMFC-instilled rabbit. nCMFC (■), nCMFC-instilled rabbit. *n* = 6–8. *^1^ *p* < 0.05, vs. Saline for each category. *^2^ *p* < 0.05, vs. CRT-solution for each category. *^3^ *p* < 0.05, vs. mCMFC for each category. The IOP-reducing effect of carteolol was remarkably enhanced by its combination with MH nanoparticles.

Figure 5 shows the effects of the instillation of CMFC on enhanced IOP in rabbit. The IOP-reducing effect was similar for CRT-solution and mCMFC, and the effects were observed for approximately 210 min after instillation. On the other hand, the combination of MH nanoparticles significantly enhanced the IOP-reducing effect, with the IOP being lower even 300 min after instillation.

## 3. Discussion

Carteolol is a nonselective β-adrenoceptor antagonist that is used extensively to treat glaucoma and ocular hypertension [24,25], although prolonged and repeated usage of topical carteolol may have adverse effects, such as causing asthma or corneal damage. These adverse effects can be improved by reducing the dose. However, carteolol is water-soluble, making it difficult to penetrate the hydrophobic cornea epithelium (low bioavailability). Therefore, improvements in the low bioavailability of carteolol are expected. We previously found that the transcorneal penetration of a water-soluble drug (timolol) could be enhanced by the co-instillation of MH nanoparticles [23], and this result suggested that the combination of MH nanoparticles may be useful as DDS for water-soluble drugs in the ophthalmic field. In this study, we designed nCMFC, and demonstrated its ability to enhance the bioavailability and IOP-reducing effect of dissolved carteolol.

First, we prepared nCMFC by the use of additives (methylcellulose (MC), benzalkonium chloride (BAC), and d-mannitol (mannitol)) and the bead mill method. BAC was selected as a preservative, and mannitol was added to prevent the corneal toxicity of BAC [26]. MC was used to enhance the efficiency of the bead mill [20]. The mean particle size of the MH nanoparticles in nCMFC was 73.5–113.5 nm (Figure 1A,C,E), and the solubility of MH was 3.7 μg/mL in lacrimal fluid (Figure 2A). Moreover, no obstruction of the nasolacrimal duct by nCMFC was observed in normal rabbits, since the amount of lacrimal fluid remained unchanged by the repetitive instillation of nCMFC (Figure 2B). In addition, we found that both the in vivo (repetitive instillation of nCMFC for 4 weeks) and in vitro (treatment of nCMFC in HCE-T cells) studies caused no observable corneal damage (Figure 2C,D). We previously reported that MH nanoparticles do not cause obstruction of the nasolacrimal duct or corneal toxicity [23]. Therefore, these results support our previous studies, and show the safety of nCMFC.

Next, we investigated the effect of nCMFC instillation on the corneal permeability of carteolol. The combination of carteolol with MH microparticles had no effect on the corneal permeability of carteolol, since the transcorneal penetration of carteolol in corneas treated with mCMFC was similar to that in corneas treated with CRT-solution in the in vitro study (Figure 3). In contrast to the results with mCMFC, the transcorneal penetration of carteolol was increased by its combination with MH nanoparticles, and the *J*_c_, *K*_p_, and *D* in nCMFC were all significantly higher than those for CRT-solution and mCMFC (Table 1 and Figure 3). Furthermore, the addition of MH nanoparticles also enhanced the carteolol levels in the aqueous humor (AUC), and the AUC for nCMFC was 9.64-fold higher than that for the CRT-solution in the in vivo study (Table 2 and Figure 4). These results suggest that combining dissolved carteolol with MH nanoparticles may be useful for enhancing the bioavailability of carteolol.

It is known that carteolol decreases IOP by reducing the formation of aqueous humor in the ciliary body of the eye and is used as therapy for glaucoma [10,11,12,13,14]. Therefore, we also demonstrated the preventive effects of the instillation of nCMFC in experimental models of glaucoma (Figure 5). Rabbits have circadian rhythms of aqueous flow and IOP, and the IOP in rabbits is enhanced at night (dark phase) [27,28]. It has been reported that increases in cyclic adenosine monophosphate, norepinephrine, adrenergic neurotransmitter, and second messenger for β-adrenergic signal transduction in the aqueous humor are related to the enhancement of IOP in the dark phase, and that experimental models for glaucoma that take advantage of the circadian rhythm of normal rabbits are suitable for evaluating the relevance of reducing IOP in human eyes [27,28]. Therefore, we used experimental rabbit models in this study. The IOP in normal rabbits was 12.7 ± 0.4 mmHg (*n* = 7), and the IOP was increased to 21.5 ± 0.5 mmHg (*n* = 20) by keeping the rabbits in a dark room for 5 h. Both CRT-solution and mCMFC decreased the enhanced IOP, and the IOP-reducing effect was similar for both formulations. nCMFC also decreased the IOP, and the attenuation was significantly greater in comparison with rabbits receiving CRT-solution or mCMFC (Figure 5). In addition, the IOP-reducing effect was lost approximately 210 min after the instillation of CRT-solution or mCMFC while, in contrast, the IOP in rabbits instilled with nCMFC remained lower even 300 min after instillation. This extension in the effective time may allow a reduction in the application frequency, resulting in an attenuation of the adverse effects, such as asthma and corneal damage. On the other hand, it is known that the effects of carteolol 2% eye drops are similar to those of timolol 0.5% eye drops in lowering daytime IOP in open-angle glaucoma [29,30,31]. The IOP-reducing effect of commercially available timolol 0.5% eye drops was observed for 210 min after instillation, and the maximum IOP-reducing effect was 4.6 mmHg at 60 min (*n* = 6). In this way, the IOP-reducing effect of nCMFC containing 1% carteolol is higher than that of commercially available timolol 0.5% eye drops. From these results, it is possible that the dose of carteolol can be reduced by combining the drug with MH nanoparticles, and this lower dose should reduce the side effects. The instillation of nCMFC may provide effective therapy for glaucoma patients.

**Scheme 1:**
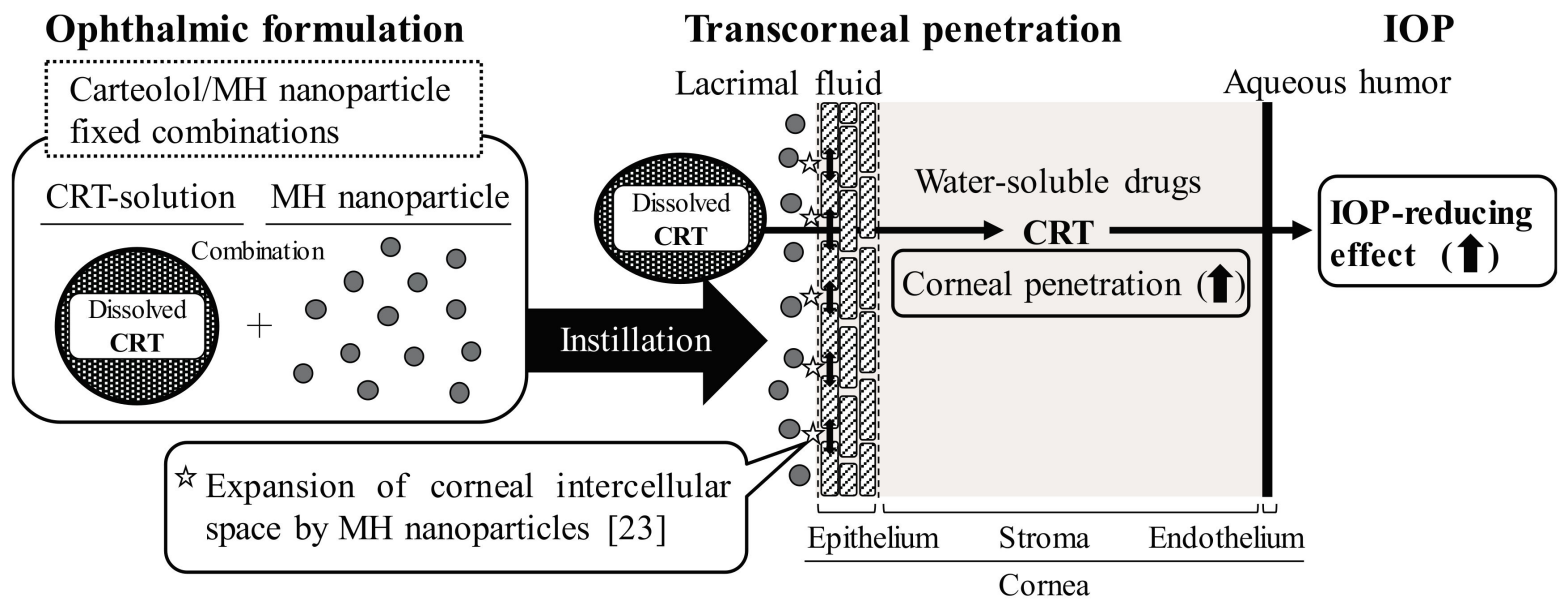
Mechanism for enhancement of transcorneal penetration and IOP-reducing effect by the combination of carteolol and MH nanoparticles.

It is important to elucidate the mechanism by which the corneal permeability of carteolol is enhanced by its combination of MH nanoparticles. Diebold and Calonge [32] reported that nanoparticles (size < 100 nm) enhance the passage of large water insoluble-molecules through the cornea. Our previous reports also showed that the *J*_c_, *K*_p_, and *D* values for drug solid nanoparticles are higher than those for microparticles [22]. Moreover, the instillation of MH nanoparticles widens the corneal intercellular space, and enhances the *J*_c_, *K*_p_, and *D* of timolol (water-soluble drug) [23]. Taken together, we hypothesize that the ratio of the corneal intercellular space increases by treatment with MH nanoparticles, resulting in an enhancement in the uptake of dissolved carteolol (Scheme 1).

However, it is not sufficiently clear for an ophthalmic DDS using nanoparticles. Therefore, further studies are needed to elucidate the precise mechanism for the enhancement of carteolol transcorneal penetration by combining it with MH nanoparticles. In addition, it is important to clarify any changes in the corneal surface that may be caused by nanoparticle treatment. Therefore, we next plan to investigate the effect of MH nanoparticles on tight junctions in corneal cells, and measure the zeta potential data and electron microscopy image (TEM and SEM) in nCMFC.

## 4. Materials and Methods

### 4.1. Materials

MH, mannitol, MC, and the Magnesium B test kit were provided by Wako Pure Chemical Industries, Ltd. (Osaka, Japan). Carteolol and Benoxil (0.4%) were purchased from Tokyo Chemical Industry Co., Ltd. (Tokyo, Japan) and Santen Pharmaceutical Co., Ltd. (Osaka, Japan), respectively. BAC was obtained from Kanto Chemical Co., Inc. (Tokyo, Japan). Sterilized Tear Production Measuring Strips and fluorescein were provided by Showa Yakuhin Kako Co., Ltd. (Tokyo, Japan) and Alcon Japan (Tokyo, Japan), respectively. Cell Count Reagent SF was purchased from Nacalai Tesque (Kyoto, Japan). All other chemicals used were of the highest purity commercially available.

### 4.2. Animals

Japanese albino rabbits (2.5 kg) were provided from Shimizu Laboratory Supplies Co. Ltd. (Kyoto, Japan), and housed under standard conditions. All experiments were performed in accordance with the Kindai University Faculty of Pharmacy Committee Guidelines for the Care (project identification code KAPS-25-003, 1 April 2013) and the Association for Research in Vision and Ophthalmology resolution on the use of animals in research.

### 4.3. Preparation of CMFC Ophthalmic Formulations

Carteolol (1%) was dissolved in saline containing 0.5% MC, 0.001% BAC, and 0.5% mannitol (1% CRT-solution), and MH nanoparticles were prepared according to our previous study using a bead mill [23]. Briefly, MH was dispersed in saline containing 0.5% MC, 0.001% BAC, and 0.5% mannitol, and the dispersions were treated with the mead mill (MH nanoparticles). mCMFC and nCMFC were prepared by mixing the CRT-solution and MH particles (final concentrations: carteolol 1%, MH 0.01%). Table 3 shows the formulations in the CRT-solution, mCMFC, and nCMFC. For preparation, the solvents containing additives were filtered under aseptic conditions through a Minisart CE 0.20 μm (Costar, Cambridge, MA, USA). The eye drops were made isotonic, and the pH was adjusted to 8.5. The SALD-7100 (Shimadzu Corp., Kyoto, Japan, refractive index 1.60−0.10i) and the NANOSIGHT LM10 (QuantumDesign Japan, Tokyo, Japan) were used to measure particle-size (wavelength, 405 nm (blue); measurement time, 60 s; viscosity of the suspension, 0.904–0.906 mPa∙s), and SPM-9700 was used to measure the particle-image (Shimadzu Corp., Kyoto, Japan). The carteolol concentration was determined by a Shimadzu LC-20AT HPLC system (Shimadzu Corp., Kyoto, Japan) using 25 mM phosphate buffer/acetonitrile (92/8, *v*/*v*) as the mobile phase and detection at 252 nm. Other conditions were as follows: column, Inertsil^®^ ODS-3 (GL Science Co., Inc., Tokyo, Japan); column oven, 35 °C; flow rate, 0.2 mL/min.

### 4.4. Solubility of MH in Lacrimal Fluid

Lacrimal fluid was obtained from rabbits. nCMFC (50 μL) was added to 50 μL lacrimal fluid, and the amount of dissolved magnesium ion was measured using the Magnesium B test kit according to the manufacturer’s instructions.

### 4.5. Measurement of Lacrimal Fluid and Corneal Toxicity by Instillation of nCMFC

Thirty microliters of nCMFC was instilled into the eyes of rabbits twice a day (9:00 and 19:00) for 4 weeks. The Schirmer’s test was used to measure the amount of lacrimal fluid using Sterilized Tear Production Measuring Strips, and the wound area was stained with 1% fluorescein and measured using a TRC-50X fundus camera (Topcon, Tokyo, Japan) equipped with a digital camera. In addition, the in vitro corneal toxicity was also measured by using human cultured corneal epithelial cells (HCE-T cells) (23). The HCE-T cells (1 × 10^4^ cells) were stimulated by 1% CRT-solution, mCMFC, or nCMFC for 0–60 s in 96-well microplates (IWAKI, Chiba, Japan). After that, Cell Count Reagent SF was added, and the absorbance (Abs) at 490 nm was measured. The cell viability was calculated according to the manufacturer’s instructions, as represented by Equation (1):

Cell viability (%) = Abs_treatment_/Abs_non-treatment_ × 100 (1)

### 4.6. In Vitro Transcorneal Penetration of CMFC

The experiment was performed according to our previous reports using a methacrylate cell [20]. Carteolol concentrations were determined by HPLC as described above. The carteolol penetration rate (*J*_c_), penetration coefficient through the cornea (*K*_p_), cornea/preparation partition coefficient (*K*_m_), diffusion constant within the cornea (*D*), and lag time (*τ*) were analyzed using the obtained data and Equations (2)–(4):

(2) $$\tau =\frac{\delta^{2}}{6D}$$

(3) $$J_{\text{c}}=\frac{K_{\text{m}}\cdot D\cdot C_{\mathrm{CRT}}}{\delta }=K_{\text{p}}\cdot C_{\mathrm{CRT}}$$

(4) $$Q_{t}=J_{\text{c}}\cdot A\cdot \left(t-\tau \right)$$

where *A* (0.78 cm^2^) is the effective area of the cornea; *C*_CRT_ is the carteolol concentration; *Q_t_* is the total amount of carteolol appearing in the reservoir solution at time *t*; and *δ* (6.25 × 10^−2^ cm) is the thickness of the cornea [20].

### 4.7. In Vivo Transcorneal Penetration of CMFC

The experiment was performed according to our previously reported method [23]. Thirty microliters of CMFC was instilled into the eyes of rabbits, and samples of the aqueous humor (5 μL) were collected. The carteolol concentrations were determined by HPLC as described above, and the AUC and the MRT were calculated from the obtained data [23].

### 4.8. Measurement of Intraocular Pressure in Rabbits

The IOPs were monitored at intervals of 30 min for 300 min following the instillation of 30 μL of ophthalmic formulation containing carteolol using a TonoPen XL (Medtronic SOLAN, Jacksonville, FL, USA). IOP enhancement in rabbits was induced by keeping them in a darkroom for 5 h (10:00–15:00) [17].

### 4.9. Statistical Analysis

Unpaired Student’s *t*-test, Aspin-Welch’s *t*-test, or Dunnett’s multiple comparison was used, and *p* < 0.05 was considered significant. All data are expressed as the mean ± S.E.M. (standard error of the mean).

## 5. Conclusions

We designed nCMFC, and showed that high levels of dissolved carteolol can be delivered into the aqueous humor by its instillation into the eyes of rabbits. Moreover, the repetitive instillation with nCMFC for 4 weeks (twice/day) resulted in no observable corneal stimulation. The combination of MH nanoparticles may make it possible to enhance the corneal penetration of water-soluble drugs, and the findings concerning this DDS may be useful for developing anti-glaucoma eye drugs and therapy for patients with glaucoma.

## Figures and Tables

**Table 1:** Pharmacokinetic analysis of carteolol in the in vitro transcorneal penetration of nCMFC.

Formulation	*J*_c_ (nmol·cm^−2^·min^−1^)	*k*_p_ (×10^−4^·min^−1^)	*k*_m_ (×10^−3^)	τ (min)	*D* (×10^−5^·cm^2^·min^−1^)
CRT-solution	9.7 ± 1.2	6.4 ± 0.8	1.0 ± 0.2	17.1 ± 2.1	3.8 ± 0.2
mCMFC	9.9 ± 1.3	6.0 ± 1.0	1.1 ± 0.2	16.7 ± 1.8	3.9 ± 0.6
nCMFC	17.6 ± 1.7 *^1,2^	11.6 ± 1.1 *^1,2^	0.2 ± 0.1 *^1,2^	1.9 ± 0.4 *^1,2^	33.1 ± 4.7 *^1,2^

mCMFC, carteolol/MH microparticles fixed combination. nCMFC, carteolol/MH nanoparticles fixed combination. The parameter data were analyzed by Equations (2)–(4). *n* = 7. *^1^ *p* < 0.05, vs. CRT-solution for each category. *^2^ *p* < 0.05, vs. mCMFC for each category.

**Table 2:** Pharmacokinetic analysis of carteolol in the in vivo transcorneal penetration of nCMFC.

Formulation	AUC (mM·min)	MRT (min)
CRT-solution	0.98 ± 0.14	65.6 ± 0.5
mCMFC	1.65 ± 0.23	62.7 ± 3.1
nCMFC	9.45 ± 1.71 *^1,2^	60.6 ± 2.3

mCMFC, carteolol/MH microparticles fixed combination. nCMFC, carteolol/MH nanoparticles fixed combination. AUC, area under the carteolol concentration-time curve. *MRT*, mean residence time. *n* = 7. *^1^ *p* < 0.05, vs. CRT-solution for each category. *^2^ *p* < 0.05, vs. mCMFC for each category.

**Table 3:** Formulation in CRT-solution, mCMFC, and nCMFC.

Formulation	MH Particles	Carteolol	MC	BAC	Mannitol	Treatment
CRT-solution	－	1%	0.5%	0.001%	0.5%	－
mCMFC	0.01%	1%	0.5%	0.001%	0.5%	－
nCMFC	0.01%	1%	0.5%	0.001%	0.5%	Bead mill

mCMFC, carteolol/MH microparticles fixed combination. nCMFC, carteolol/MH nanoparticles fixed combination. MC, methylcellulose. BAC, benzalkonium chloride.

## Abbreviations

Abs	absorbance
AUC	area under the carteolol concentration-time curve
BAC	benzalkonium chloride
carteolol	carteolol hydrochloride
CMFC	carteolol/MH nanoparticle fixed combinations
DDS	drug delivery systems
IOP	intraocular pressure
mannitol	d-mannitol
MC	methylcellulose
MH	magnesium hydroxide
*MRT*	the mean residence time
S.E.M.	standard error of the mean

## References

[1] American Academy of Ophthalmology (2010). Primary Open-Angle Glaucoma Preferred Practice Patterns.

[2] National Institute for Health and Care Excellence (2009). Glaucoma: Dianosis and Management of Chronic Open Angle Glaucoma and Ocular Hypertension.

[3] Boland M.V., Ervin A.M., Friedman D., Jampel H., Hawkins B., Volenweider D., Chelladurai Y., Ward D., Suarez-Cuervo C., Robinson K.A. (2012). Treatment for Glaucoma: Comparative Effectiveness.

[4] Boland M.V., Ervin A.M., Friedman D.S., Jampel H.D., Hawkins B.S., Vollenweider D., Chelladurai Y., Ward D., Suarez-Cuervo C., Robinson K.A. (2013). Comparative effectiveness of treatments for open-angle glaucoma: A systematic review for the U.S. Preventive Services Task Force. Ann. Intern. Med..

[5] Dong Z.M., Wollstein G., Schuman J.S. (2016). Clinical Utility of Optical Coherence Tomography in Glaucoma. Investig. Ophthalmol. Vis. Sci..

[6] Scripsema N.K., Garcia P.M., Bavier R.D., Chui T.Y., Krawitz B.D., Mo S., Agemy S.A., Xu L., Lin Y.B., Panarelli J.F. (2016). Optical Coherence Tomography Angiography Analysis of Perfused Peripapillary Capillaries in Primary Open-Angle Glaucoma and Normal-Tension Glaucoma. Investig. Ophthalmol. Vis. Sci..

[7] Janczewski P., Boulanger C., Iqbal A., Vanhoutte P.M. (1988). Endothelium-dependent effects of carteolol. J. Pharmacol. Exp. Ther..

[8] Man I.A.V., Schalekamp M.A. (1982). How intrinsic sympathomimetic activity modulates the haemodynamic responses to beta-adrenoreceptor antagonists: A clue to the nature of their antihypertensive mechanism. Br. J. Clin. Pharmacol..

[9] Stewart W.C. (1994). Carteolol, an ophthalmic beta-adrenergic blocker with intrinsic sympathomimetic activity. J. Glaucoma.

[10] Li T., Lindsley K., Rouse B., Hong H., Shi Q., Friedman D.S., Wormald R., Dickersin K. (2016). Comparative Effectiveness of First-Line Medications for Primary Open-Angle Glaucoma: A Systematic Review and Network Meta-analysis. Ophthalmology.

[11] Montanari P., Marangoni P., Oldani A., Ratiglia R., Raiteri M., Berardinelli L. (2001). Color Doppler imaging study in patients with primary open-angle glaucoma treated with timolol 0.5% and carteolol 2%. Eur. J. Ophthalmol..

[12] Altan-Yaycioglu R., Turker G., Akdol S., Acunas G., Izgi B. (2001). The effects of beta-blockers on ocular blood flow in patients with primary open angle glaucoma: A color Doppler imaging study. Eur. J. Ophthalmol..

[13] Tamaki Y., Araie M., Tomita K., Tomidokoro A., Nagahara M. (1997). Effects of topical adrenergic agents on tissue circulation in rabbit and human optic nerve head evaluated with laser speckle tissue circulation analyzer. Surv. Ophthalmol..

[14] Tamaki Y., Araie M., Tomita K., Nagahara M., Tomidokoro A. (1997). Effect of topical beta-blockers on tissue blood flow in the human optic nerve head. Curr. Eye Res..

[15] Nagai N., Deguchi S., Otake H., Hiramatsu N., Yamamoto N. (2017). Therapeutic Effect of Cilostazol Ophthalmic Nanodispersions on Retinal Dysfunction in Streptozotocin-Induced Diabetic Rats. Int. J. Mol. Sci..

[16] Nagai N., Yoshioka C., Tanabe W., Tanino T., Ito Y., Okamoto N., Shimomura Y. (2015). Effects of Ophthalmic Formulations containing Cilostazol Nanoparticles on Retinal Vasoconstriction in Rats Injected with Endothelin-1. Pharm. Anal. Acta.

[17] Okamoto N., Ito Y., Nagai N., Murao T., Takiguchi Y., Kurimoto T., Mimura O. (2010). Preparation of Ophthalmic Formulations Containing Cilostazol as an Anti-glaucoma Agent and Improvement in Its Permeability through the Rabbit Cornea. J. Oleo Sci..

[18] Nagai N., Mano Y., Ito Y. (2016). An Ophthalmic Formulation of Disulfiram Nanoparticles Prolongs Drug Residence Time in Lens. Biol. Pharm. Bull..

[19] Nagai N., Yoshioka C., Mano Y., Tanabe W., Ito Y., Okamoto N., Shimomura Y. (2015). A nanoparticle formulation of disulfiram prolongs corneal residence time of the drug and reduces intraocular pressure. Exp. Eye Res..

[20] Nagai N., Ito Y., Okamoto N., Shimomura Y. (2014). A nanoparticle formulation reduces the corneal toxicity of indomethacin eye drops and enhances its corneal permeability. Toxicology.

[21] Nagai N., Nakazawa Y., Ito Y., Kanai K., Okamoto N., Shimomura Y. (2017). A Nanoparticle-Based Ophthalmic Formulation of Dexamethasone Enhances Corneal Permeability of the Drug and Prolongs Its Corneal Residence Time. Biol. Pharm. Bull..

[22] Nagai N., Ono H., Hashino M., Ito Y., Okamoto N., Shimomura Y. (2014). Improved corneal toxicity and permeability of tranilast by the preparation of ophthalmic formulations containing its nanoparticles. J. Oleo Sci..

[23] Nagai N., Ogata F., Otake H., Kawasaki N., Nakazawa Y., Kanai K., Okamoto N., Shimomura Y. (2017). Co-instillation of nano-solid magnesium hydroxide enhances corneal permeability of dissolved timolol. Exp. Eye Res..

[24] Kawai M., Nagaoka T., Takahashi A., Sato E., Yoshida A. (2012). Effects of topical carteolol on retinal arterial blood flow in primary open-angle glaucoma patients. Jpn. J. Ophthalmol..

[25] Henness S., Swainston H.T., Keating G.M. (2007). Ocular carteolol: A review of its use in the management of glaucoma and ocular hypertension. Drugs Aging.

[26] Nagai N., Yoshioka C., Tanino T., Ito Y., Okamoto N., Shimomura Y. (2015). Decrease in Corneal Damage due to Benzalkonium Chloride by the Addition of Mannitol into Timolol Maleate Eye Drops. J. Oleo Sci..

[27] Liu J.H., Dacus A.C., Bartels S.P. (1991). Adrenergic mechanism in circadian elevation of intraocular pressure in rabbits. Investig. Ophthalmol. Vis. Sci..

[28] Kiuchi Y., Yoshitomi T., Gregory D.S. (1992). Do alpha-adrenergic receptors participate in control of the circadian rhythm of IOP?. Investig. Ophthalmol. Vis. Sci..

[29] Mirza G.E., Karakucuk S., Temel E. (2000). Comparison of the effects of 0.5% timolol maleate, 2% carteolol hydrochloride, and 0.3% metipranolol on intraocular pressure and perimetry findings and evaluation of their ocular and systemic effects. J. Glaucoma.

[30] Stewart W.C., Shields M.B., Allen R.C., Lewis R.A., Cohen J.S., Hoskins H.D., Hetherington J.N., Bahr R.L., Noblin J.E., Delehanty J.T. (1991). A 3-month comparison of 1% and 2% carteolol and 0.5% timolol in open-angle glaucoma. Graefes Arch. Clin. Exp. Ophthalmol..

[31] Stewart W.C., Cohen J.S., Netland P.A., Weiss H., Nussbaum L.L. (1997). Efficacy of carteolol hydrochloride 1% vs. timolol maleate 0.5% in patients with increased intraocular pressure. Nocturnal Investigation of Glaucoma Hemodynamics Trial Study Group. Am. J. Ophthalmol..

[32] Diebold Y., Calonge M. (2010). Applications of nanoparticles in ophthalmology. Prog. Retin. Eye Res..

